# High-Temperature Properties of Hot Mix Asphalt Modified with Different Nanomaterials

**DOI:** 10.3390/nano15241845

**Published:** 2025-12-08

**Authors:** Yousuf M. Hamed AlHamdo, Amjad H. Khalil Albayati, Mazen J. Al-Kheetan

**Affiliations:** 1 Civil Engineering Department, University of Baghdad, Baghdad 10070, Iraq; yousif.hamed2301@coeng.uobaghdad.edu.iq (Y.M.H.A.); a.khalil@uobaghdad.edu.iq (A.H.K.A.); 2 Civil and Environmental Engineering Department, College of Engineering, Mutah University, Karak 61710, Jordan

**Keywords:** asphalt, nanomaterial, MSCR, DIC, permanent deformation, VESYS

## Abstract

Rutting is a predominant distress in asphalt pavements, particularly in hot climatic regions. This study systematically investigated the high-temperature performance of hot mix asphalt modified with five nanomaterials, namely, nano-silica (NS), nano-alumina (NA), nano-titanium (NT), nano-zinc (NZ), and carbon nanotubes (CNTs), under consistent laboratory conditions. Modification dosages were selected up to 10% for NS, NA, and NT, and up to 5% for NZ and CNTs. The experimental methodology comprised the following: (i) binder rheological characterization through rotational viscosity, G*/sinδ, and multiple stress creep recovery (MSCR) to quantify rutting susceptibility; (ii) chemical and microstructural assessments using Fourier transform infrared spectroscopy (FTIR) and scanning electron microscopy (SEM); (iii) mixture-level evaluation via repeated-load axial testing coupled with digital image correlation (DIC) to monitor permanent microstrain evolution; and (iv) rutting performance over a 20-year period using the VESYS 5W predictive model. A cost–performance analysis was further incorporated to assess the economic viability of each nanomaterial. The results demonstrated that nanomodification substantially improved rutting resistance, consistent with reductions in non-recoverable creep compliance and permanent microstrain. Among additives, the 8% NS mixture exhibited the most favorable performance, maintaining a present serviceability index (PSI) of 2.5 after 20 years, whereas the un-modified mixture dropped below the failure threshold within a few years. These findings confirm that nanomaterial selection and dosage can meaningfully enhance the structural and performance of asphalt pavements.

## 1. Introduction

Permanent deformation, commonly known as rutting, is a significant issue in asphalt pavements, characterized by longitudinal depressions formed by repeated traffic loading, particularly at elevated temperatures. This distress compromises safety, reduces driving comfort, and increases maintenance costs. A 1.1-inch rut depth, for example, causes a decrease in the flexible pavement serviceability index from 4.2 to 2.5, highlighting the critical impact of rutting on pavement performance [1]. High temperatures exacerbate rutting by softening the asphalt binder, reducing its ability to resist deformation, and causing the accumulation of non-recoverable strain in the asphalt mixture [2,3]. To mitigate these issues, recent research has focused on improving the high-temperature performance of asphalt concrete (AC) by incorporating nanomaterials.

Defined by the European Union Observatory for Nanomaterials (EUON) as particles ranging from 1 to 100 nm in size, nanomaterials offer substantial benefits due to their exceptionally high surface area and minimal required dosage [4,5,6]. When used within optimal dosage ranges, nanomaterials generally contribute to higher binder stiffness and greater rutting resistance [7]. Nanomaterials enhance rutting resistance by improving binder rheology, increasing stiffness and elasticity, and enhancing the asphalt’s ability to recover from deformation [8,9,10,11]. Additionally, they reinforce the asphalt matrix at the nano-scale, improving its structural integrity under repeated load cycles [12,13]. Nanoparticles also enhance adhesion between the binder and aggregates, which helps distribute traffic load more effectively [14,15].

Nanomaterials are also reported to improve asphalt in other aspects, such as indirect tensile strength [16,17]. Nanomaterials likewise are found to enhance the resilient modulus, split tensile strength, fracture energy, and fatigue life while also reducing moisture susceptibility [18,19,20]. Adhesion between the asphalt binder and the aggregate may also be intensified by nanomodification [21].

Numerous studies have demonstrated that nanomaterials can significantly improve the rheological, mechanical, and high-temperature resistance of asphalt binders and mixtures. For instance, nano-silica (NS), nano-alumina (NA), and nano-titanium (NT) have been reported to reduce rut depth, increase Marshall stability, and enhance performance grade [22,23,24,25,26,27,28,29], while nano-zinc (NZ) and carbon nanotubes (CNTs) improve thermal stability and rutting resistance even at low dosages [30,31,32,33,34].

Despite these promising results, conflicting findings exist on the effects of these nanomaterials on asphalt properties, particularly high-temperature performance and rutting resistance. Inconsistencies include variation in reported optimal dosages; for instance, previous studies report effectiveness at low dosages (CNTs at 0.5%) [34], while others required higher contents (2%) [31]. Also, a broad optimum dosage range of 4% to 15% was observed in previous studies for NS modification [23,24,35], which could largely be attributed to differences in material properties, mixing approaches, and evaluation criteria. Differences in how researchers test and evaluate nanomaterials often lead to performance outcomes that are not directly comparable. What constitutes an optimum dosage can also vary depending on the chosen criteria, with some studies prioritizing cost-effectiveness. Furthermore, even when adequate initial dispersion is obtained, nanoparticles may undergo re-agglomeration during storage [36]. Some even stated that nano-zinc, for example, may not contribute to performance improvement, but rather improves UV ray protection [14], while others reported contradictory findings. The same applies to different studied nanomaterials. This raises doubts about whether dosage thresholds are material-specific or influenced by test conditions. The opposite of the common belief is that improvements in binders and mixes are not always comparable, as an improvement in a binder does not always translate into the same performance in the mixture. Also, variations in test conditions and asphalt grade among studies lead to contradictory results in the literature. Moreover, in the majority of studies, individual nanomaterials are examined on their own, and it is hard to compare them to each other in terms of their effectiveness. This highlights the necessity of comparative studies, in a systematic manner, with similar conditions. Further, there is little research on which digital image correlation (DIC) technique is used to observe the distributions of strain fields and a performance prediction program based on nano-modified asphalt mixtures.

This study aims to mitigate these variations by critically assessing the impact of various nanomaterials in various doses on the physical and rheological characteristics of asphalt binders and asphalt mixtures, with a special emphasis on performance in terms of high temperatures and rutting resistance. This research aims to determine the optimal levels of nanomaterial modification that enhance the durability and resilience of asphalt pavements under high-temperature conditions. The standardization of mixing approaches, dose optimization criteria, and performance testing would improve the consistency and reproducibility of inter-study results and facilitate the practical application of nanomaterials in pavement engineering practice. This comparative analysis aims to bridge existing knowledge gaps and support the establishment of practical guidelines for the application of nanomaterials in pavement engineering.

## 2. Materials

### 2.1. Asphalt Binder

The asphalt binder used in this research was obtained from the Al-Daurah refinery, with the physical and rheological properties shown in Table 1 and Table 2.

### 2.2. Aggregates

**Figure 1:**
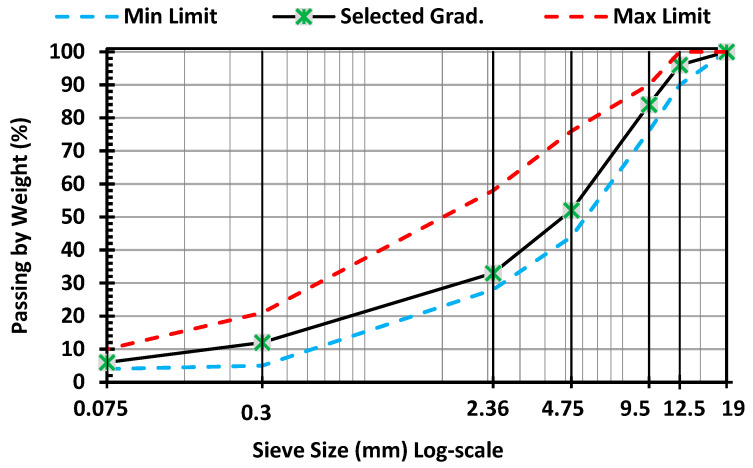
Aggregate gradation.

A dense graded hot mix asphalt mixture (Type D5) was prepared following the gradation and design criteria specified in ASTM D3515 [44]. Limestone dust with a specific gravity of 2.70 was used as filler. The aggregate gradation is shown in Figure 1, and its properties are listed in Table 3.

### 2.3. Nano-Modifiers and Mixing

Five modifiers were added to the asphalt binder from Hebei Suoyi New Material Technology Co., Ltd., Hebei, Handan, China, with the properties shown in Table 4.

Twenty-six different binder formulations were prepared for NS, NT, and NA at 2%, 4%, 6%, 8%, and 10%, and for NZ and CNTs at 1%, 2%, 3%, 4%, and 5% (plus a control with 0%). The modification dosages are by weight of asphalt binder. The selected nanomaterial dosages in this study were chosen to reflect ranges commonly investigated in the literature while enabling a systematic evaluation of performance at low, medium, and high contents. The modification dosages were selected based on the previous literature, as shown in Table 5.

**Figure 2:**
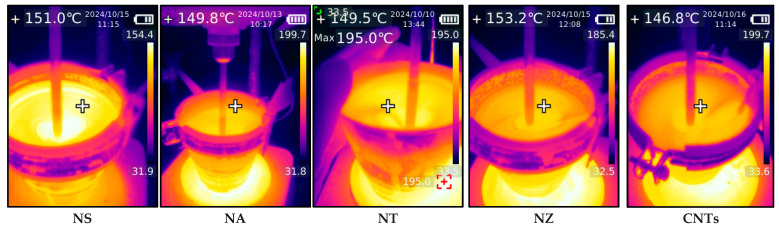
Mixing of nanomaterials.

The dry blending method was used to disperse each modifier into the asphalt binder, with an initial mixing at 400 rpm and a 4 g/min addition rate, followed by 3000 rpm for 20 min at 150 ± 5 °C, based on previous research contributions [16,51,58,59,60]. Meanwhile, the temperature was monitored using a thermal camera with (256 × 192 pixel) thermal resolution to ensure adequate mixing and homogeneity, as illustrated in Figure 2. The designations include the nanomaterial name and then the dose; for instance, NS-6 indicates 6% nano-silica by weight of asphalt binder, and so on.

## 3. Experimental Tests

**Figure 3:**
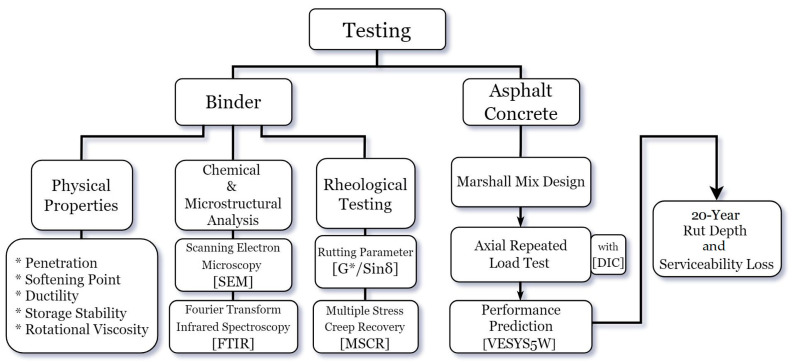
Experimental program.

The experimental framework employed is outlined in Figure 3.

### 3.1. Physical Properties of Asphalt Binder

It is important to note that traditional binder tests are empirical in nature and are intended to provide general indicators of binder consistency and temperature susceptibility; they do not represent fundamental rheological properties nor directly predict field performance. Therefore, some degree of non-linearity between dosage and test response is expected, particularly when modification alters the microstructure or surface interactions rather than bulk thermal behavior [61,62].

#### 3.1.1. Penetration

The penetration depth of a standard needle (25 °C, 100 g, 5 s) according to AASHTO T 49 [37] was tested to assess binder consistency; a lower penetration indicates a harder binder, which usually correlates with better high-temperature rutting resistance.

#### 3.1.2. Softening Point

The ring and ball test was conducted according to AASHTO T 53 [39], which determines the temperature at which the binder softens and flows. A higher softening point implies improved stiffness at high service temperatures, thus indicating better rutting performance.

#### 3.1.3. Ductility

The ductility test was conducted according to AASHTO T 51 [41]. The binder’s elongation capacity was evaluated at an intermediate temperature. This ensures that, while stiffness increases with additives, the binder still retains adequate flexibility.

#### 3.1.4. Storage Stability

The storage stability of modified asphalt binders was evaluated according to ASTM D7173 [63] to assess the uniform dispersion of nanomaterials during thermal storage. The blended binder was poured into vertically positioned aluminum tubes and stored at 163 °C for 48 h. After cooling, the samples were sectioned into top and bottom thirds, and the softening point of each section was measured.

#### 3.1.5. Rotational Viscosity

Rotational viscosity (two replicates) was measured according to AASHTO T 316 [64] to determine the binder’s viscosity at mixing and compaction temperatures (135 and 165 °C) and assess the workability and stiffness of the nano-modified asphalt binder. Nanomaterials often increase binder viscosity, which can enhance rutting resistance by making the binder stiffer, but it must remain within workable ranges for paving operations.

### 3.2. Chemical and Microstructural Analysis

#### 3.2.1. Fourier Transform Infrared Spectroscopy (FTIR)

**Figure 4:**
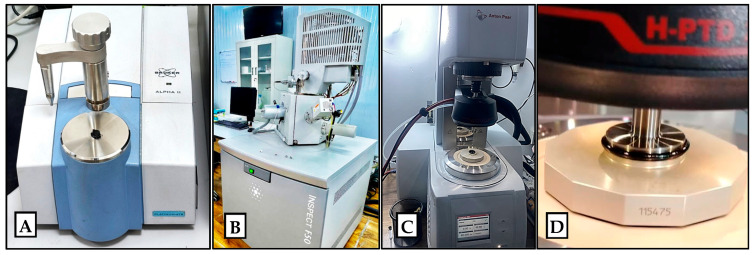
(**A**) Fourier transform infrared spectroscope and (**B**) scanning electron microscope. (**C**) Dynamic shear rheometer apparatus and (**D**) multiple stress creep recovery test.

The test was performed using a Bruker Alpha II spectrometer (Bruker Optics GmbH, Ettlingen, Germany) (Figure 4A) to identify functional groups and assess chemical changes in the asphalt binders. Spectra were collected over 400–4000 cm^−1^ with a resolution of 4 cm^−1^, and the transmittance mode was used for all measurements. The analysis focused on characteristic peaks, such as carbonyl (C=O) and sulfoxide (S=O), to ensure the nanomodification did not significantly accelerate aging, monitor the interaction between the nanomaterials and the binder, and detect any chemical changes or new functional groups resulting from the addition of nanomaterials. While many nanoparticles (such as SiO_2_, TiO_2_, and Al_2_O_3_) are inorganic and may not form new chemical bonds with the hydrocarbon binder, FTIR can confirm whether any additives cause reactions or alter oxidative aging characteristics.

#### 3.2.2. Scanning Electron Microscopy (SEM)

Microstructural analysis was conducted using a Field Emission Scanning Electron Microscope (FE-SEM), model Inspect F50 by FEI Company (FEI Company, Hillsboro, OR, USA) (Figure 4B), to examine the surface morphology and microstructure of the nano-modified asphalt binders. The samples were analyzed under high vacuum with appropriate magnification to assess the dispersion quality and interaction of nanomaterials within the binder matrix. Binder samples were examined to observe the dispersion of nanoparticles within the asphalt matrix. SEM imaging can reveal whether nanoparticles agglomerate or are evenly distributed. Well-dispersed nanoparticles can create a more uniform network, contributing to stiffness and rutting resistance [65].

### 3.3. Rheological Testing

#### 3.3.1. Rutting Parameter (G*/Sinδ)

Using Anton Paar MCR 102 (Anton Paar GmbH, Graz, Austria) Dynamic Shear Rheometer (DSR) according to AASHTO T 315 [66] and AASHTO M320 [43], the high-temperature Superpave rutting parameter (G*/sin δ) was obtained for each binder at suitable temperatures (64 °C and above). According to Superpave specifications, a higher G*/sin δ for an unaged binder indicates better resistance to rutting. This parameter was used to determine how each nanomaterial and dosage affected the binder’s high-temperature grade.

#### 3.3.2. Multiple Stress Creep Recovery (MSCR)

The MSCR test was conducted using an Anton Paar MCR 102 rheometer (Anton Paar GmbH, Graz, Austria) (Figure 4C) (25 mm plate, 1 mm gap, RTFO-aged sample) in accordance with AASHTO T 350 [67]. The test was conducted (2 test replicates) at a representative high pavement temperature (70 °C) to measure the binder’s creep and recovery under repeated loading. In the MSCR test (Figure 4D), a low non-recoverable creep compliance (Jnr) yields better rutting resistance, and a high percent recovery (%R) indicates superior rutting resistance. Each binder was subjected to creep loads of 0.1 kPa and 3.2 kPa. The MSCR results (Jnr and %R) provided insights into how the nanoparticle modifications improved the binder’s ability to recover and resist permanent deformation under traffic loading.

### 3.4. Marshall Mix Design

**Figure 5:**
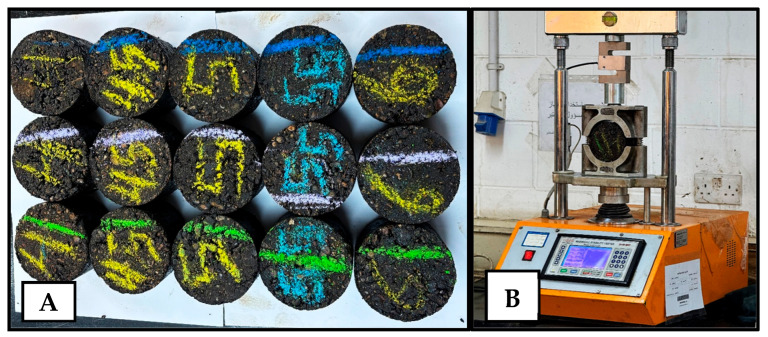
(**A**) Marshall specimens and (**B**) Marshall stability and flow apparatus.

The Marshall mix design method, standardized in AASHTO T 245 [68], was employed to determine the optimal binder content. Specimens (three replicates) were compacted according to the mix–compaction plot (158–164 °C for mixing and 149–153 °C for compaction temperatures) and tested for Marshall stability, flow, and key volumetric properties. This method provided the basis for testing the nano-modified asphalt concrete on mixture strength, stiffness, and overall mix quality. The Marshall specimens and test apparatus are shown in Figure 5.

### 3.5. Axial Repeated Loading Test

**Figure 6:**
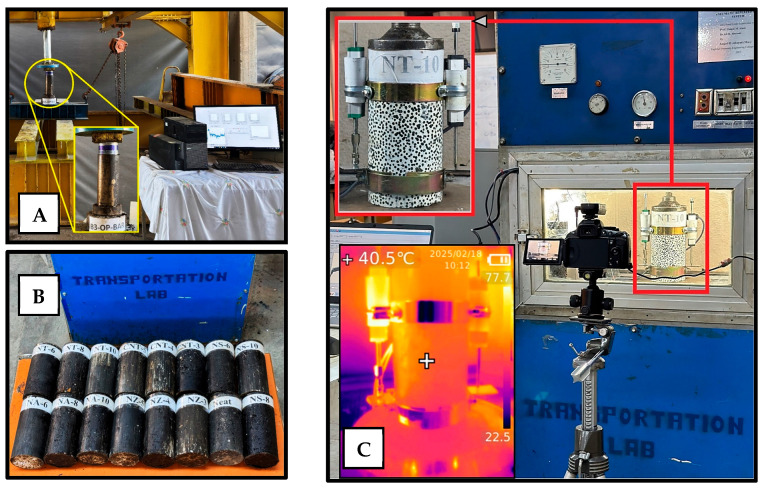
(**A**) Example 8 × 4 in specimen preparation, (**B**) 8 × 4 in specimens, and (**C**) repeated load apparatus.

Optimizing the asphalt content for each mix containing nanomaterials would introduce two varying parameters simultaneously: modification and asphalt content. This would lead to uncertainty in attributing performance differences solely to nanomaterial modification. Thus, the optimum asphalt content (OAC) was kept constant. The test specimens included 8″ × 4″ cylindrical AC specimens (2 replicates), prepared according to the Marshall mix design parameters, using the compressive dual plunger method, with a load of 45–50 tons (metric) held for 1–2 min to achieve the target air voids. The specimens are shown in Figure 6A–C. In this test, in accordance with BS EN 12697-25:2005 [69], a cyclic load (simulating repetitive wheel loading) was applied axially at 40 °C, as measured by a thermal probe inside a dummy specimen in the test chamber. Each specimen was subjected to a repeated loading sequence of 60 psi applied for 0.1 s, followed by a 0.9 s rest period, to simulate traffic loading conditions. The accumulation of permanent strain was measured (by means of 2 linear variable distance transducers (LVDTs) 180° apart with a gauge length of 100 mm) over thousands of cycles to determine the mixture’s permanent deformation susceptibility. The repeated loading test was conducted for up to 10,000 cycles or terminated earlier (specimen failure). Subsequently, a power law model (Equation (1)) (Monismith et al. 1975 [70] and Barksdale 1972 [71]) was fitted to the log–log accumulated permanent strain data with cycles to determine the slope and intercept parameters, which characterized the rate and magnitude of permanent deformation.

(1) $$\varepsilon_{p}=I{\;*\;(N)}^{S}$$

where

ε_p_ = Permanent strain.

N = No. of stress application. 

I = Intercept coefficient.

S = Slope coefficient.

### 3.6. Digital Image Correlation (DIC)

**Figure 7:**
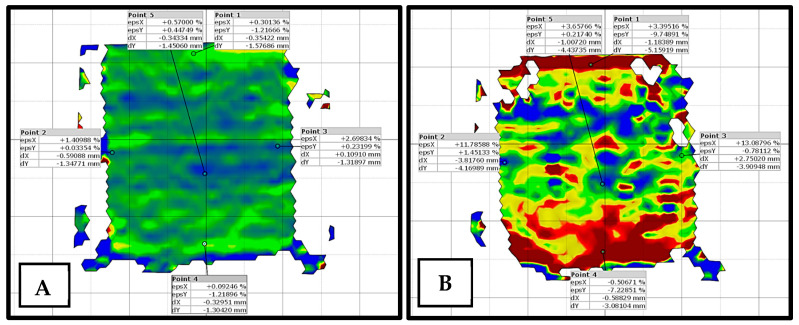
(**A**) 6% nano-titanium specimen and (**B**) 6% nano-titanium specimen after 10,000 load cycles.

A crucial aspect of the repeated loading experiment is the use of DIC to visualize strain development on the specimen surface. DIC is an optical technique that tracks the deformation of a speckle pattern applied to a specimen, providing full-field strain maps. DIC analysis was performed to identify where and how strains localize as the test progresses (for one of the replicated specimens). The DIC results are beneficial for comparing how the modified binders delay or reduce localized strain accumulation. The DIC setup consisted of a 16 mp digital camera (Nikon D5100; Nikon Corp., Tokyo, Japan) with a 50 mm lens placed in front of the specimen during the test (≈2.8 h), with a constant 20 s time interval between each image taken. The specimens were painted white with a random speckle pattern, including 2–3 mm black dots, after which the images were analyzed with Zeiss Inspect Correlate 2025 software (Carl Zeiss GOM Metrology GmbH, Braunschweig, Germany). Figure 7 highlights a test sample (NT-6) after analysis.

### 3.7. Twenty-Year Performance Modeling

**Figure 8:**
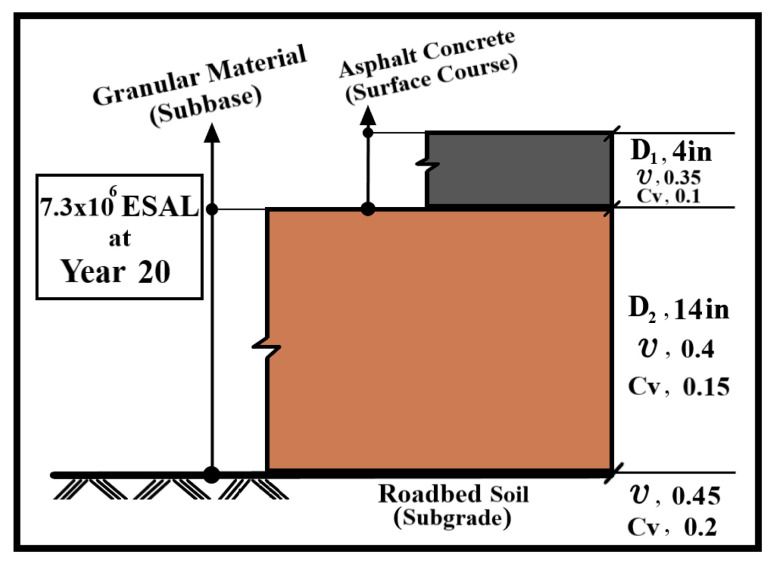
Pavement layer properties.

To translate the laboratory findings into projected field performance, a mechanistic pavement performance model was applied. A viscoelastic pavement simulation program (VESYS 5W, version 5W-1.0.1; Federal Highway Administration (FHWA), Washington, DC, USA) was used to predict rut depth growth and the Present Serviceability Index (PSI) over 20 years for pavements incorporating the modified binders. The modeled pavement structure consisted of a 100 mm (4 in.) asphalt layer (using the tested mix), underlain by a 350 mm (14 in.) subbase and a subgrade. Poisson’s ratio and the coefficient of variation for each layer are shown in Figure 8. The permanent deformation characteristics obtained from the repeated load test were used to derive the rutting rate parameters (μ and α) required by VESYS 5W, as expressed in Equations (2) and (3).

(2) α=1−Slope

(3) $$\mu =\frac{Slope\times Intercept}{Resilient\;Microstrain}$$

The traffic loading assumptions included 7.3 million repetitions of an 18-kip equivalent single axle load (ESAL) with 87 psi tire pressure, under a 40 °C climatic condition. The model outputs rut depth and decline in PSI. Since PSI is a widely accepted indicator of ride quality that decreases as rutting and surface roughness increase, the failure criteria were defined following AASHTO design guides as PSI falling below 2.5 [1] or rut depth exceeding 12.5 mm [72].

### 3.8. Cost–Performance Analysis

Nanomaterial prices vary widely across regions and suppliers, reflecting differences in purity, particle size, and surface treatment. For instance, nano-silica prices range from USD 90 to USD 1650 per kg, and organically modified grades can be even more expensive [73]. In contrast, the nano-silica used in this study was sourced at approximately USD 26/kg, which is substantially lower than the values that used to be typically reported. This difference reflects the improved availability of nanomaterials [28] and has a direct positive impact on economic feasibility by significantly reducing the material cost component of nano-modified binders. Consequently, while regional price variations may influence cost margins, the overall conclusion favoring low-cost, high-performance nanomaterials remains robust. This is further supported by a cost reduction of up to 33% (as reported in previous studies) for nano-modified binders compared with polymer-modified binders [74].

The cost–performance analysis of nanomaterial-modified asphalt binders provides an essential perspective on the balance between mechanical improvement and economic feasibility. To compare performance across materials, the improvement relative to the neat binder was calculated using Equation (4):

(4) $$Improvement\;\left(\%\right)=\;\frac{Jnr\;neat-Jnr\;modified}{Jnr\;neat}\;\times 100$$

Equation (4) was used to represents the percentage reduction in Jnr, which is a standard way of expressing relative improvement, following the standard MSCR-based approach. Additionally, the normalized cost-effectiveness was determined by dividing the improvement per 1% dosage by the cost of incorporating 1% nanomaterial (USD/ton of binder). This approach enables the identification of the most cost-efficient nanomaterial on a per-unit basis. For benchmarking, the normalization in Formula (5) was applied:

(5) $$Normalized\;Value=\;\frac{X_{max}-X}{X_{max}-X_{min}}$$

where *X* represents the Jnr value of the modified binder and *X_max_* and *X_min_* are the maximum and minimum Jnr values observed, respectively.

Equation (5) was adopted because it provides a consistent and unbiased way to compare rutting performance among binders whose Jnr values lie on different numerical scales. This approach preserves performance ranking, enables combination with cost metrics, and avoids the bias that would occur if raw Jnr values were used. Min–max normalization is widely recommended in engineering decision-making and material-selection frameworks [75,76].

## 4. Results and Discussion

### 4.1. Physical Properties of Asphalt Binder

#### 4.1.1. Penetration

**Figure 9:**
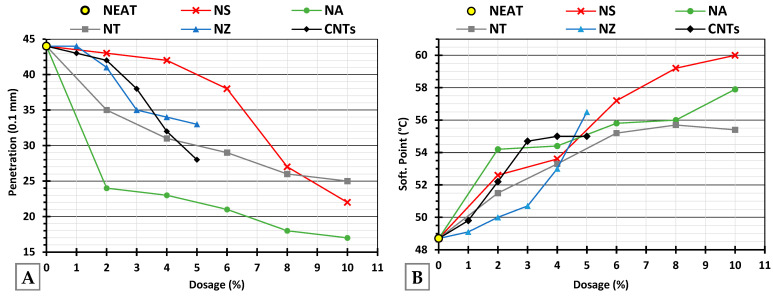
(**A**) Penetration of modified binder and (**B**) softening point of modified binder.

A decreasing trend in penetration (Figure 9A) was observed with increasing modifier dosage, suggesting that the incorporation of nanomaterials enhanced binder stiffness. This trend aligns with the known stiffening effect of nanomaterials. NZ showed moderate reductions; NA caused a significant and consistent drop, even at 2%, which will be discussed in the following sections. NT and CNTs showed an almost linear reduction. NS showed a relatively higher penetration than the others at the same dosage, indicating a weaker stiffening effect.

#### 4.1.2. Softening Point

As shown in Figure 9B, NS exhibited a steep, progressive increase, reaching 60 °C at 10%, indicating excellent high-temperature performance. NA peaked at 58 °C and then slightly stabilized. NT and CNTs stabilized at 6% and 3%, respectively. NZ illustrated the lowest overall rise at lower dosages.

#### 4.1.3. Ductility

**Figure 10:**
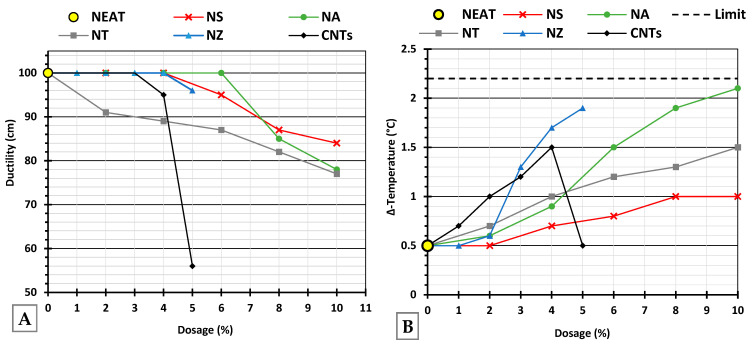
(**A**) Ductility of the modified binder and (**B**) storage stability of the modified binder at multiple dosages.

Increasing the dosage of most of the nanomaterials led to somewhat reduced ductility at higher dosages, reflecting a loss of flexibility due to increased stiffness. NS, NA, and NT exhibited comparable trends, as illustrated in Figure 10A, indicating a consistent stiffening effect that progressively limited the binder’s elongation capacity. NZ maintained ductility above 95 cm up to 5%, demonstrating excellent flexibility retention and making it highly suitable where elasticity and crack resistance are priorities. As for CNTs, ductility remained stable until 4% and then dropped sharply at 5%, suggesting a critical threshold beyond which flexibility is compromised.

#### 4.1.4. Storage Stability

Figure 10B presents the temperature difference between the top and bottom portions of the conditioned samples after thermal storage. A ΔT below 2.2 °C (dashed line) is generally considered acceptable, ensuring the modifier remains uniformly dispersed and does not settle or segregate. The nano-modified binders exhibited some increase in ΔT compared to the neat binder, varying by nanomaterial type and dosage. NS and NT demonstrated a stable performance across most dosages, remaining well below the 2.2 °C limit. NA showed a steady increase in ΔT, approaching the stability limit at the 10% dosage, indicating moderate susceptibility to separation at higher dosages. NZ resulted in a steeper increase by 5%NZ and may exceed the threshold at higher dosages. CNTs displayed nonlinear behavior at 5%, with a reduction, suggesting possible over-saturation, such that sedimentation is improbable due to the fibrous morphology and higher density.

#### 4.1.5. Rotational Viscometer (RV)

**Figure 11:**
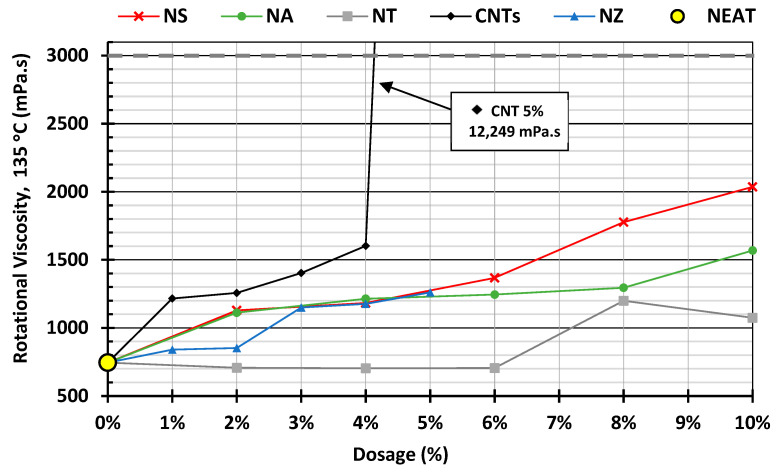
Rotational viscosity of modified binder.

**Figure 12:**
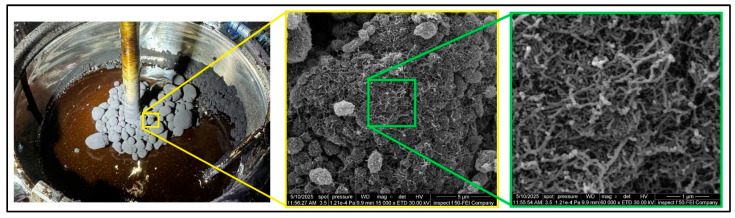
Carbon nanotube (5%) initial dispersion.

As shown in Figure 11, NS exhibited a steady and significant increase, indicating a strong thickening effect due to the porous structure, exceptional specific surface area, and strong surface interactions. NA showed a moderate, linear rise, suggesting a good balance between modification and workability. NT behaved more like an inert filler up to moderate dosages, resulting in minimal viscosity change until particle–particle interactions become dominant (lower viscosity overall). NZ remained relatively stable across dosages, with a slight increase, making it a steady, workable modifier. CNTs with their fibrous one-dimensional extremely high aspect ratio, readily entangle and agglomerate, forming networks that strongly resist flow, and make uniform dispersion within the asphalt binder very difficult, indicating severe processing challenges. This explains the sharp spike in viscosity at 5% CNTs, which cannot be attributed to particle size alone but rather to shape-induced clustering and poor dispersion stability. This effect was most evident at 5% CNTs. During the initial mixing, the binder appeared oversaturated with CNTs, leading to network formation and a dramatic increase in viscosity. Such behavior (Figure 12) was not observed at lower dosages.

The divergent viscosity trends among the nano-modifiers can be rationalized by their interactions with SARA fractions. High-area oxides (especially NS) preferentially adsorb maltenes (aromatics/resins) [77], reducing peptization and effectively increasing the asphaltene volume fraction, which raises viscosity and rheological properties. NA exhibited the same maltene adsorption mechanism, but to a lesser extent. NT behaved filler-like up to 6%, with viscosity rising only at higher dosages through possible particle networking. Nano-zinc oxide exhibited mild adsorption with possible antioxidative effects, resulting in minimal viscosity change while still enhancing high-temperature stiffness.

Carbon nanotubes (being one-dimensional and graphitic) can interact with aromatics/asphaltenes. The extended graphitic surfaces of CNTs attract each other via Van Der Waals interactions, leading to bundling and agglomeration [78]. These interactions improve load transfer and binder stiffness, but they also trap maltenes, leading to a sharp increase in viscosity at a high CNT content. CNTs form asphaltene networks that strongly resist flow, explaining the sharp RV spike despite all particles having “nano” dimensions. Thus, while CNTs can significantly enhance rutting resistance, their strong noncovalent interactions also make dispersion a significant challenge.

Based on the rotational viscosity measurements, it was determined that proceeding with higher nanomaterial dosages was justified, as the associated increase in binder stiffness is expected to enhance resistance to permanent deformation.

### 4.2. Chemical and Microstructural Analysis

#### 4.2.1. Fourier Transform Infrared Spectroscopy (FTIR)

**Figure 13:**
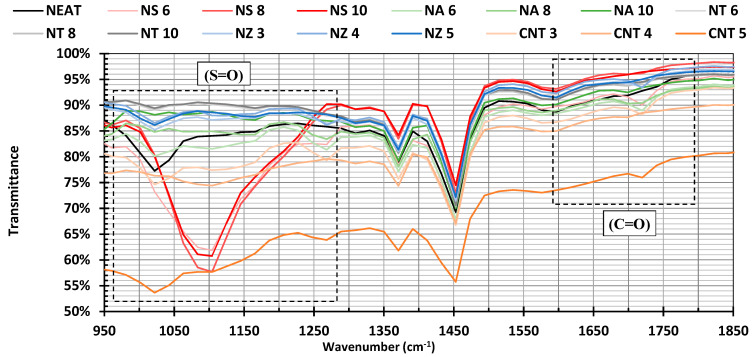
Fourier transform infrared spectroscopy (FTIR) results.

The oxidative aging resistance of the asphalt binders modified with various nanomaterials was assessed by calculating oxidation indices for two key functional groups: sulfoxide (S=O) and carbonyl (C=O). Figure 13 shows that the absorbance peaks at 1020 cm^−1^ (sulfoxide), 1270 cm^−1^ (secondary sulfoxide), and 1720 cm^−1^ (carbonyl), and these indices are shown in Table 6.

The neat binder had the maximum content of sulfoxide and a fairly high carbonyl index. When nanomaterial was incorporated, different magnitudes of decrease in these oxidation indices were seen, which is an indication that the modifiers affected oxidative stability.

NZ and CNTs showed slight to moderate changes and slight decreases in the sulfoxide indices. Although CNTs decreased the sulfoxide index, the carbonyl index slightly increased, indicating that structural improvement, but not chemical oxidation inhibition, can happen.

NT demonstrated the highest antioxidative activity, and both the sulfoxide and carbonyl indices were slightly decreased with further increases in dosage. NT produced sulfoxide and carbonyl indices of 0.0224 and 0.0103 at 10%, which is less than half of that in the neat binder. These findings are in line with the established radical-scavenging and UV-blocking properties of NT, making it very efficient in inhibiting oxidative aging. It was shown that the addition of nano-alumina in asphalt mixtures enhances the performance of high-temperature mixtures in terms of stiffness and reduction in permanent deformation. This is mainly because the nano-alumina particles physically reinforce it, as opposed to the chemical aging processes [26]. In addition, a previous study reported an increase in thermal conductivity and rheological/mechanical properties at high and intermediate temperatures. The improvements occurred without any major oxidizing reactions, and this implies that the improvements were due to a physical interaction between the nano-alumina and the asphalt matrix [79]. In this manner, the decrease in the values of penetration in NA-modified asphalt binders (Figure 9A) can be explained by physical mechanisms. Nano-alumina particles may be used as stiff fillers in the asphalt matrix, which limit the flow of asphalt molecules and create a firmer, more uniform binder structure, resulting in a stiffer binder.

Concerning NS modification, it showed values similar to the neat binder for the sulfoxide index. However, there was a significant decrease in the carbonyl index at higher doses up to 0.0062, which showed that it was partly resistant to oxidation. Nevertheless, on closer inspection, it is necessary to add that NS has a high siloxane (Si-O-Si) stretching peak around 1100 cm^−1^ that partially overlaps or distorts the baseline along the area of the sulfoxide index at 1030 cm^−1^. The spectral interference may cause an exaggeration of the sulfoxide index of NS-modified binders, especially at high dosages. It is worth noting that increases in both absorption bands correspond to the formation of more polar oxygenated compounds within the asphalt binder. From a compositional perspective, this shift indicates a transformation of lighter fractions (saturates and aromatics) into heavier fractions (resins and asphaltenes), which is consistent with the oxidative aging process. Such chemical changes lead to a stiffer, more brittle material that is more susceptible to cracking and durability loss. Therefore, reductions in these oxidation indices after nanomaterial modification can be interpreted as a preservation of the lighter fractions and a retardation of the aging-driven shift toward resins and asphaltenes.

#### 4.2.2. Scanning Electron Microscopy (SEM)

##### Nanomaterials

**Figure 14:**
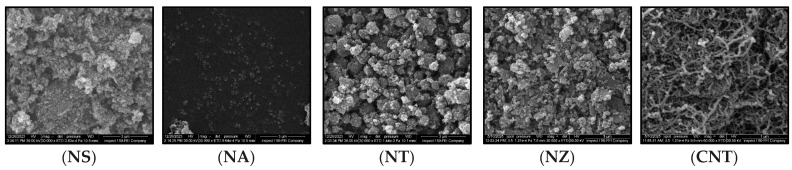
Scanning electron microscopy images of nanomaterials.

SEM images of the nanomaterials are shown in Figure 14. NS showed an irregular, agglomerated, and porous morphology, with fine particles forming loose clusters. This high surface area and rough texture contribute to strong interfacial interactions with asphalt, promoting matrix stiffening. NA appeared as well-dispersed, nearly spherical nanoparticles with a significantly smaller particle size compared to NS and NT. The uniformity and smooth morphology enhance its potential to act as a filler and to integrate homogenously in the binder matrix. NT exhibited a dense, crystalline, and granular morphology, with larger aggregated clusters than NA. The rough surfaces may increase mechanical interlocking with binder components, but they require good dispersion to be effective. NZ revealed a compact, granular structure with irregularly shaped particles and surface roughness, indicating a high surface area beneficial for mechanical interlocking and binder interaction. In contrast, CNTs showed a distinctive entangled fibrous network. This structure suggests potential for forming a reinforcing mesh within the asphalt matrix, promoting crack-bridging and strain distribution, although it may also introduce dispersion challenges if not well integrated.

##### Modified Asphalt Binder

**Figure 15:**
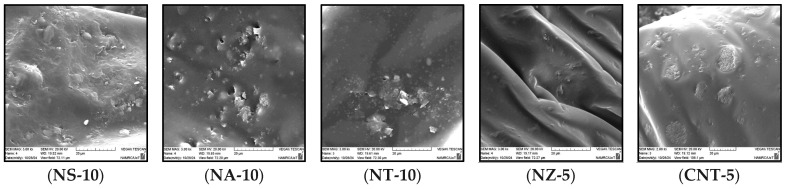
Scanning electron microscopy images of nano-modified asphalt binders.

**Figure 16:**
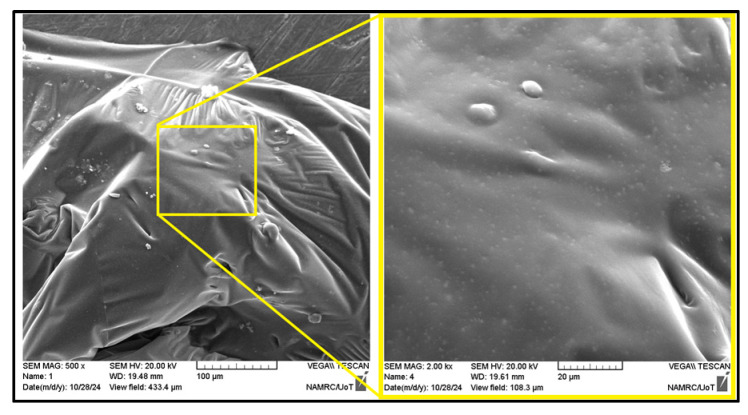
Scanning electron microscopy of 6% nano-titanium asphalt binder.

SEM imaging was conducted on the highest nanomaterial dosages selected for each modifier (as indicated in Figure 15) to capture the most pronounced microstructural effects, evaluate particle dispersion at critical concentrations, and assess potential agglomeration or network formation under maximum conditions. The microstructure of NS appeared densified and rugged, with silica particles possibly embedded within the matrix. The coarse, interwoven texture implied greater rigidity and physical support. Nevertheless, there is a possibility of local agglomeration due to some clustering. The NA-modified asphalt contained dispersed and embedded particles that had a smoother surrounding matrix. The white spots are discrete, which are alumina particles. The SEM images of the NT-6 modified binder (Figure 16) show that the matrix is mainly smooth and compact with localized spherical protrusions that are ascribed to nano-titanium clusters. The morphology is applicable in the explanation of the minimal change in viscosity to 6% since the nanoparticles are used as inert fillers, other than as a complete reinforcing network. These findings are consistent with what was mentioned by Shafabakhsh and Ani [80]. In NT-10, coarse particle clusters were observed in the binder, with a clear contrast, indicating poor blending in certain regions. While NT provides stiffness, its large particle clusters may cause localized stress points if not properly dispersed. The surface was smooth with few observable inclusions, and this suggests that it was either well dispersed or did not interact much. In the case of NZ, the absence of prominent particles indicated that microstructural reinforcement was limited, and this is in line with the lower influence of NZ on penetration and stiffness. The CNT-modified binder had clear protrusions and circular structures, which can be considered CNT-saturated zones or agglomerates. These structures imply the incomplete development of the networks, which proves the assumption of mechanical reinforcement through entanglement. It is known that nanoparticles, including carbon nanotubes, will agglomerate and separate in part when mixed with asphalt binders. Here, the nanoparticles were added in their undispersed state through high shear mixing, which led to acceptable but incomplete dispersion of the nanoparticles, as verified by SEM. In the literature, surface modification methods have been reported to improve the compatibility of nanoparticle binders and decrease phase separation, including chemical functionalization. While such approaches were not adopted in this work, future research could investigate surface-modified nanomaterials to improve interaction with asphalt binders and achieve more uniform reinforcement.

### 4.3. Rheological Testing

#### 4.3.1. Rutting Parameter (G*/Sinδ)

**Figure 17:**
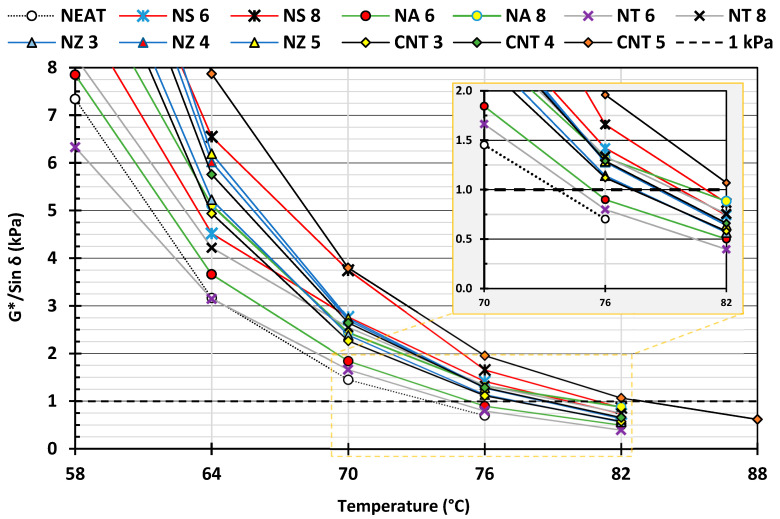
Rutting parameter of nano-modified asphalt binders.

The data in Figure 17 indicate that all the binders showed a decreasing G*/Sin δ with increasing temperature, as expected due to the softening of the asphalt. The nano-modified binders exhibited significantly higher G*/sin δ values than the neat binder across the temperature range, confirming enhanced stiffness and rutting resistance. NS-8 exhibited the second-highest G/sin δ across most temperatures, suggesting superior rutting resistance. The NA-modified binders also performed well, but they were slightly lower than NS at high temperatures. The NT binders showed moderate improvement over the neat biner, with G*/sin δ values above 1.0 kPa up to 76 °C. NZ showed a dose-dependent improvement. However, its performance is closer to the lower range of the modified binders, indicating limited stiffening capability. CNT-5 matched or exceeded NS-8 at specific points. High values across the entire range, even approaching 2 kPa at 70 °C, confirm outstanding elastic and stiffening effects. The zoomed section emphasizes performance near the critical 1.0 kPa limit. CNTs and NS were perhaps the most effective at enhancing rutting resistance, maintaining G*/sin δ values above the Superpave threshold, even at elevated temperatures. NA and NT also improved binder performance, albeit to a lesser extent.

#### 4.3.2. Multiple Stress Creep Recovery (MSCR)

**Figure 18:**
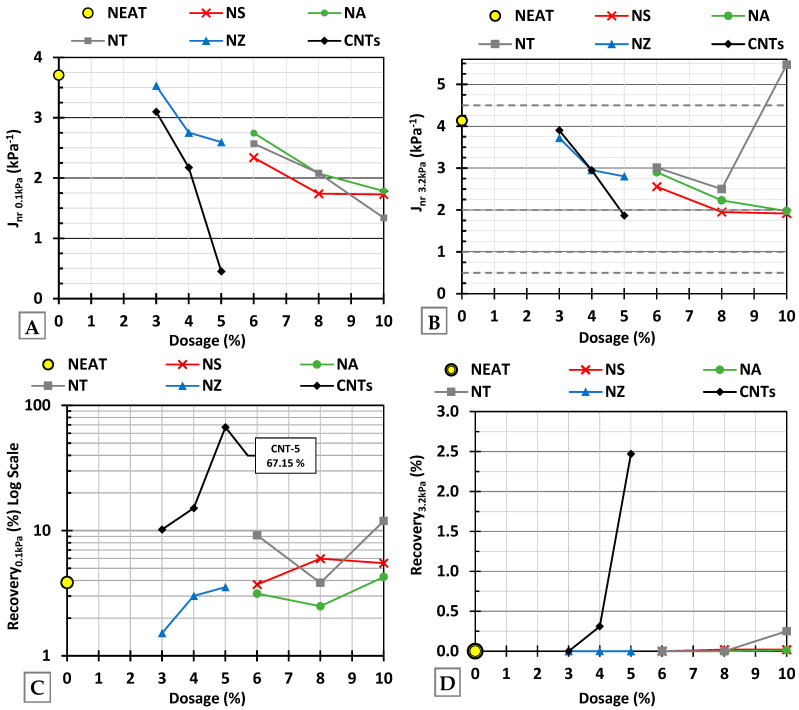
(**A**) Non-recoverable creep compliance (0.1 kPa), (**B**) non-recoverable creep compliance (3.2 kPa), (**C**) percent recovery (0.1 kPa), and (**D**) percent recovery (3.2 kPa).

The MSCR test is a more comprehensive predictor of rutting performance, especially in modified asphalt binders. At 0.1 kPa (Figure 18A), the neat binder exhibited the highest Jnr value. All nanomaterials reduced Jnr with increasing dosage, confirming improved rutting performance. NS, NA, and NT showed converging values (1.5–2.0 kPa^−1^) at higher dosages, reflecting a moderate but consistent improvement. CNTs showed the most significant improvement, with Jnr falling below 0.5 kPa^−1^ at 5%, indicating excellent performance. NZ showed the least reduction, suggesting limited stiffening efficiency. In Figure 18B, the dotted horizontal lines represent performance thresholds of J_nr3.2kPa_ (namely, S, H, V, and E). At J_nr3.2kPa_, under high stress, the trends remained consistent, but the difference between modifiers became more evident. NS and NA maintained stability with relatively low J_nr3.2kPa_, while NT-10 showed a drastic, sharp increase, suggesting instability or reduced performance at higher stress levels. CNTs again showed the lowest J_nr3.2kPa_, maintaining resistance even under heavy traffic conditions.

For % recovery, NS and NA offered limited elastic recovery, indicating a predominantly stiffening effect, as illustrated in Figure 18C,D. NT showed reasonable performance at moderate dosages but became inconsistent at high doses, with rising J_nr_ and variable recovery, possibly due to dispersion challenges or agglomeration. NZ yielded somewhat modest improvements in stiffness and minimal recovery. CNTs exhibited the most balanced and superior performance, combining low J_nr_ with high %recovery across both stress levels. This indicates both high stiffness and significant elasticity, making CNT-modified binders, under the right conditions, suitable for high-traffic or heavy-load applications. The MSCR results exhibited good repeatability, with variability across replicates below 5%. It should be noted that these results are on a binder scale and will be evaluated for asphalt concrete in the following sections.

### 4.4. Cost–Performance Analysis

**Figure 19:**
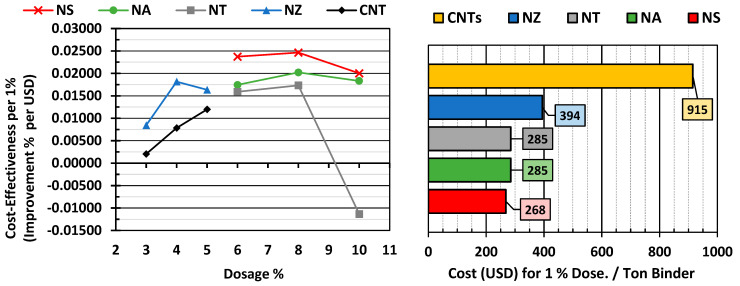
Cost−effectiveness per 1% improvement and cost per ton of binder.

It is evident that CNTs achieved the greatest Jnr gains. However, the high cost of CNTs (USD 91.5/kg) substantially reduces their cost-effectiveness, with an improvement-per-dollar ratio far lower than that of the other nanomaterials, as shown in Figure 19. In contrast, nano-silica (NS) demonstrated the best balance between performance and cost (0.02465% improvement per dollar). Similarly, nano-alumina (NA) exhibited competitive performance. Although NZ’s performance was inferior to that of NS and NA, its cost-effectiveness remained reasonably high at low dosages due to its intermediate material cost. On the other hand, nano-titanium (NT) exhibited inconsistent results. The cost–performance analysis shows that while CNTs deliver the strongest absolute performance improvements, their high cost limits their practicality for large-scale pavement applications. In contrast, NS at the 8% dosage emerges as the most cost-effective modifier. These findings emphasize the importance of considering both mechanical performance and economic viability in selecting nanomaterials for asphalt modification.

### 4.5. Marshall Mix Design

**Figure 20:**
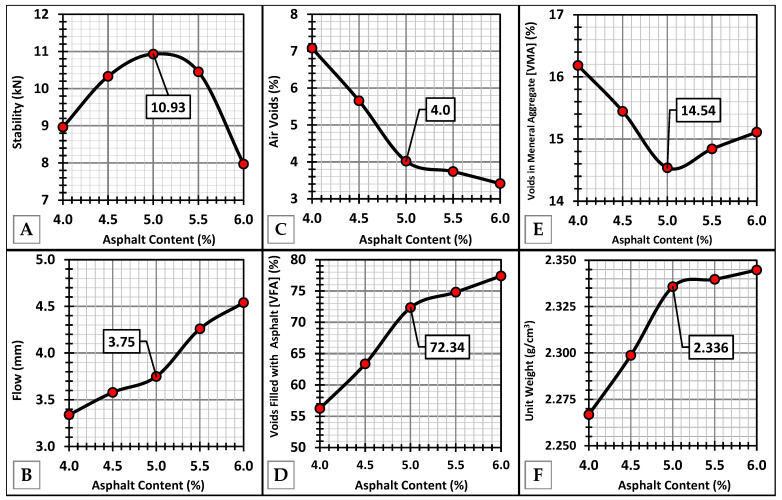
Marshall mix design properties, asphalt content and: (**A**) Stability, (**B**) Flow, (**C**) Air Voids, (**D**) Voids filled with asphalt, (**E**) Voids in mineral aggregate, and (**F**) Unit weight.

The Marshall mix design parameters are presented in Figure 20. The graphs indicate that a 5.0% asphalt binder content offers the most balanced mix properties, where strength, compactness, and durability indicators align. At this point, the mixture achieves peak stability, minimal voids, and favorable volumetric and flow characteristics, suggesting it is the optimum asphalt content (OAC). According to the Asphalt Institute MS-2 [81], the mixture satisfies heavy traffic (>10^6^ Equivalent Single-Axle Load) criteria.

### 4.6. Axial Repeated Loading Test

#### 4.6.1. Permanent Microstrain

**Figure 21:**
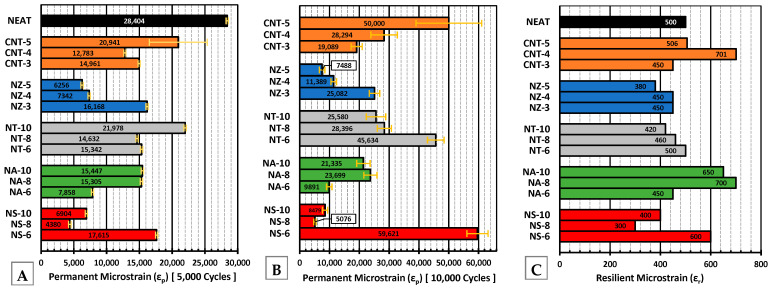
(**A**) Permanent microstrain at 5000 cycles, (**B**) permanent microstrain at 10,000 cycles, and (**C**) resilient microstrain.

Applying 60 psi of repeated axial stress resulted in a high stress level on the specimens, causing the neat asphalt mixture to fail shortly after 5000 cycles (Figure 21A). NS-8 and NZ-5 showed the lowest vertical strain, indicating excellent premature deformation resistance. NA-6 showed good performance. The CNT-modified mixes performed worse than expected when compared with the DSR results. The replicate analysis (Table 7) showed that most of the nanomaterial-modified mixtures exhibited good consistency, with coefficients of variation generally below 3%, indicating reliable repeatability between duplicate tests. Slightly higher variability was observed for NS–8 and NZ–5, though still within acceptable experimental limits. In contrast, CNT-5 showed poor reproducibility, with a coefficient of variation (COV) of about 21%, which was markedly higher than that of all the other mixes. This strong divergence underscores the inherent challenges of uniformly dispersing CNTs at higher dosages, resulting in unstable mechanical responses under repeated loading. The NT-modified mixes showed the least performance improvement compared to the other nanomaterials, slightly better than the neat mixture.

After 10,000 load cycles (Figure 21B), NS continued to outperform, particularly NS-8, and was significantly better than all the other modifiers except NZ-5 and NA-6, which yielded similar performance. NS-6, NT-10, and CNT-5 had among the highest strains, suggesting weaker long-term resistance, possibly due to dispersion or structural breakdown over time. CNTs, despite their satisfactory performance in MSCR, showed higher permanent strain, suggesting that CNTs may not form a well-dispersed reinforcing network in the asphalt concrete mix and/or at low mixing and compaction temperatures. These results emphasize the need to evaluate modifiers not only at the binder scale but also under mixture-level mechanical loading conditions.

##### One-Way ANOVA

Next, we compared the neat binder with the best-performing dosage of each modifier (i.e., NS-8, NA-6, NT-10, NZ-5, and CNT-3), focusing on the optimum dosage reflecting the most practical conditions for engineering application, using one-way ANOVA (Table 8).

The analysis of variance confirmed that the differences in permanent strain among the binders were statistically significant. The test showed that the improvements observed with nanomodification were not due to random variation but reflected a consistent effect of the additives on rutting resistance. These results provide strong evidence that nanomaterials, when used at optimal dosages, meaningfully reduce permanent deformation, underscoring their value as performance-enhancing modifiers in asphalt binder design.

#### 4.6.2. Resilient Microstrain

Resilient microstrain (Figure 21C) is inversely correlated to the resilient modulus (M_r_), a key indicator of a material’s ability to recover elastically after repeated loading. The neat asphalt exhibited one of the highest values, consistent with its high permanent deformation under repeated loading. NS-8 recorded the lowest resilient strains, signifying the highest stiffness and elastic resilience. The NZ-modified mixtures followed closely. NT, followed by NA, fell within the moderate range. The CNT-modified mixtures recorded the highest resilient microstrain values. This reflects lower elastic stiffness, even though CNTs showed high elastic recovery in the binder-level MSCR tests. This could indicate that CNTs can enhance recoverability but not the resilient modulus, likely due to imperfect dispersion and/or ineffective reinforcement within the composite matrix at the mixture level.

#### 4.6.3. Resilient Modulus (M_r_)

**Figure 22:**
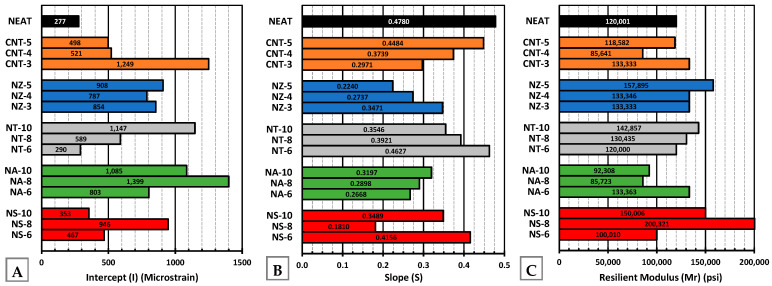
(**A**) Intercept (power model) at cycle 1, (**B**) slope of power model, and (**C**) resilient modulus.

This parameter provides a direct measure of load-bearing capacity under repeated loading. Higher values indicate resistance to nonelastic deformation, which is essential for preventing rutting in flexible pavements. The results (Figure 22C) directly align with the low resilient microstrain observed in Figure 21C. Although NT-6 showed a relatively high M_r_, its prolonged performance was less favorable (elevated permanent strain after 10,000 cycles). However, at 5000 cycles, NT-6 exhibited lower permanent deformation, suggesting that its early-stage rutting resistance was effective but less sustained under extended cyclic loading. This discrepancy may be attributed to the material’s initial stiffness providing early resistance to deformation, while microstructural fatigue or limited elastic recovery under prolonged cyclic stress could have led to accelerated strain accumulation in later stages. This highlights the importance of evaluating both short-term and long-term deformation behavior when assessing modifier effectiveness.

#### 4.6.4. Power Model Parameters

A high slope (Figure 22B) indicates a faster rate of permanent deformation (weaker rutting resistance), while a high intercept (Figure 22A) reflects a higher initial strain level, suggesting an early plastic response. Considering that the neat binder failed after just 5000 cycles, the nano-modified mixtures demonstrated a clear superior rutting resistance lasting 10,000 cycles. Among them, NS-8 emerged as the most effective, combining a low slope with a moderate intercept, resulting in slow and stable strain progression under prolonged loading. NS-8 was closely followed by NZ-5, with the second-lowest slope, showcasing its effectiveness in slowing permanent deformation accumulation.

Although NS-8 and NS-10 exhibited similar initial permanent strain levels, NS-8 showed superior long-term performance. This suggests that 8% nano-silica achieved more effective dispersion and matrix interaction, whereas 10% may have led to agglomeration or excessive stiffness, compromising durability. A similar trend was observed for NA-6 and CNT-3, where lower dosages offered a better balance between reinforcement and resistance to permanent deformation.

While improvements in G*/sin δ and Jnr are typically associated with improved resistance to deformation, the trends in this study did not consistently align across all the nanomaterials. Several modifiers exhibited notable improvements in binder rheology without producing proportional enhancements in mixture rutting performance. As such, mixture-level deformation is governed not only by stiffness and recoverability but also by aggregate structure, binder–aggregate adhesion, and the effectiveness of the nanomaterial dispersion.

### 4.7. Digital Image Correlation (DIC)

**Figure 23:**
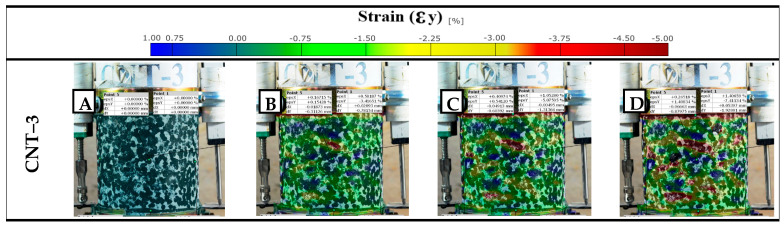
Vertical technical strain at approx. (**A**) 25%, (**B**) 50%, (**C**) 75%, and (**D**) 100% of testing.

**Figure 24:**
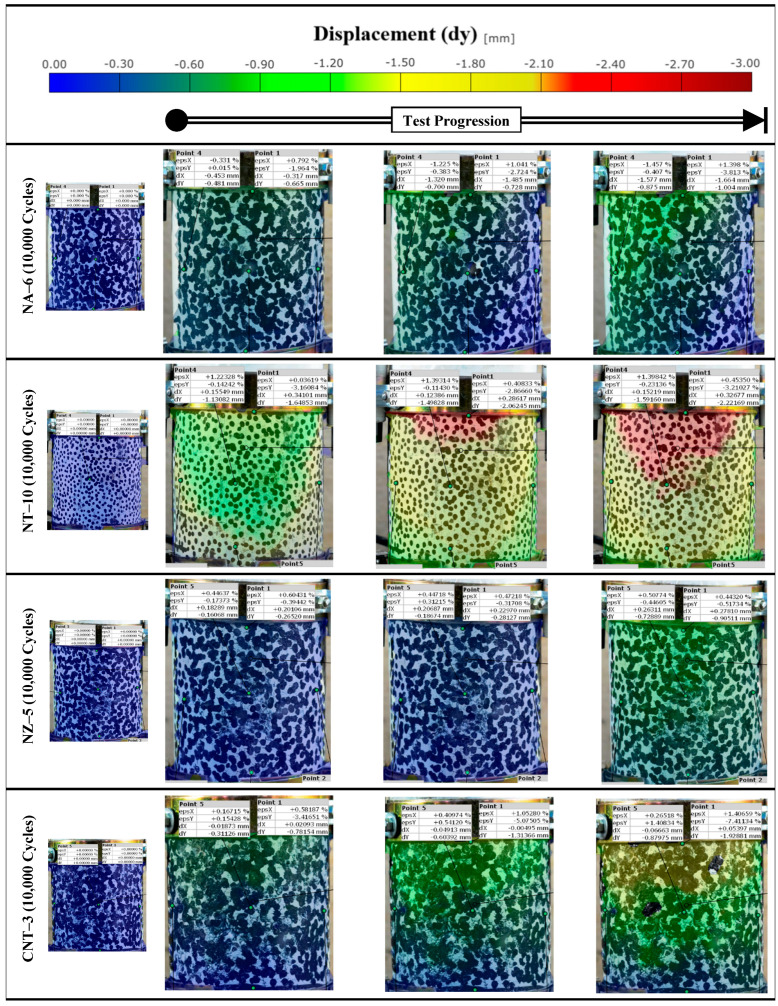
Vertical displacement with cycles.

**Figure 25:**
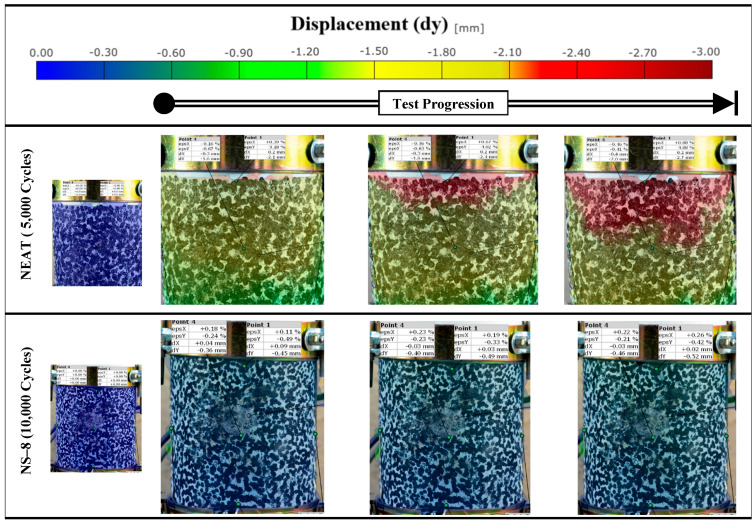
Vertical displacement with cycles.

The DIC images provided a full-field visualization of vertical displacement (dy) during the repeated loading test (for the best-performing modified mixtures), offering insights into how deformation evolves spatially and temporally within each specimen. The vertical technical strain of CNT-3 (Figure 23) showed uneven strain distribution with irregular zones suggestive of localized strain concentration, possibly due to poor dispersion or matrix inconsistencies. These visual patterns validate the mechanical outcomes and highlight the importance of optimizing nanomaterial dosage for effective deformation control in asphalt mixtures. The color scale (Figure 24 and Figure 25) ranges from blue (zero displacement) to red (high displacement), corresponding to increasing levels of vertical strain. The observations are consistent with the mechanical test data, particularly the power model slope–intercept results, further confirming the enhanced performance of the nano-modified binders. Additionally, a comparison between the DIC-derived strain and traditional LVDT measurements showed an average deviation of approximately 5%, which falls within acceptable limits for practical asphalt deformation analysis. This level of agreement supports the validity and reliability of DIC as a noncontact measurement method for capturing strain behavior during repeated loading.

### 4.8. Twenty-Year Performance Modeling (VESYS 5W)

**Figure 26:**
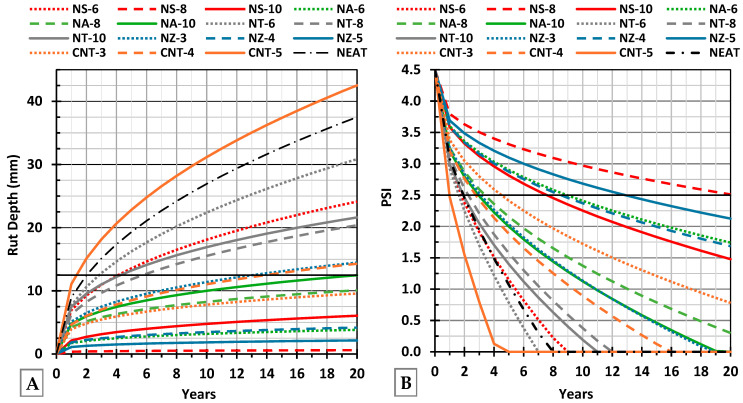
(**A**) Rut depth with years and (**B**) PSI with years.

VESYS 5W simulation showed that there is a significant disparity in the rutting behavior and pavement performance of the different asphalt mixtures. The neat binder had severe rutting, with a rut depth of up to 30 mm over time, which is a very poor criterion. CNT-5 was the most rutted of the nano-modified mixes, which also surpassed the neat binder after a few years. Conversely, NS-8 and NZ-5 did not rut deeply and were not even critical during the simulation, indicating strong long-term rutting resistance. NA-6 and CNT-3 showed moderate performance. The trend of the Pavement Serviceability Index (PSI) reflects the rutting behavior. NS-8 had the highest PSI in 20 years, which is a sign of a high-quality ride and surface integrity. The values of relatively high PSI were also maintained in NZ-5 and NA-6, which supports the fact that they are stable in terms of structural performance in the long term. Figure 26A,B shows the results of VE-SYS 5W. NZ-5 and NA-6 also preserved relatively high PSI values, confirming their stable long-term structural performance. The VESYS 5W results are shown in Figure 26A,B.

## 5. Conclusions and Recommendations

Based on the binder and mixture test results, and within the constraints of the limited materials and testing conducted, the following conclusions can be drawn:

1. All five nanomaterials (NS, NA, NT, NZ, CNTs) enhanced the high-temperature rutting resistance of asphalt binders and mixtures compared to the neat binder. This was demonstrated through improved binder stiffness, lower Jnr values, and reduced permanent deformation in mixture testing, with NS and NZ showing the most significant improvements.
2. Optimum dosages were identified as 8% NS, 6% NA, 10% NT, 5% NZ, and 3% CNTs.
3. Excessive addition led to diminishing or even adverse effects.
4. For hot-climate, heavy-traffic highways, 8% NS and 5% NZ are recommended, as they provide the highest rutting resistance and long-term serviceability. NA at 6% is suitable where a balance between stiffness and flexibility is required, while CNTs at 3% may be effective with advanced dispersion techniques. NT offered moderate benefits but was less efficient compared to the other nanomaterials.
5. The primary mechanism of improvement is physical reinforcement. Nanoparticles refine the binder microstructure, filling voids and forming a reinforcing network that resists shear deformation under load. This was supported by MSCR results showing lower non-recoverable compliance and DIC observations of more uniform strain distribution across mixtures.
6. VESYS 5W performance prediction showed that the nano-modified binders substantially delay rutting accumulation. After 20 years of simulated traffic at 40 °C, pavements with 8% NS retained a PSI above 2.5, while the un-modified pavement dropped sharply within a few years. These yields extended pavement service life, reduced maintenance intervals, and provided potential life-cycle cost savings.
7. While the findings of this study demonstrated improvements in binder and mixture-level performance, certain limitations must be acknowledged. This work was conducted at a laboratory scale, used a single binder source and content, and employed a fixed aggregate gradation under a controlled simulated climate. Field-scale pavement trials are therefore essential to validate construction practicability and long-term performance. Future research should also explore synergies between nanomaterials and other modifiers, while also assessing fatigue and low-temperature cracking behavior, and incorporate sustainability and cost–benefit analyses. In addition, extending the investigation to different material systems, climatic conditions, and full-scale engineering applications would further enhance the practical relevance of nano-modified asphalt technology.

## Figures and Tables

**Table 1:** Physical assessment of asphalt binder.

Property	Standard	Unit	Result	Limit
Penetration (25 °C, 100 g, 5 s)	AASHTO T 49 [37]	0.1 mm	44	40–50
Retained pen. after rolling thin film oven (RTFO)	AASHTO T 49 [37]/ AASHTO T 240 [38]	%	59	≥55
Softening point	AASHTO T 53 [39]	°C	48.7	-
Specific gravity (25 °C)	ASTM D 70 [40]	/	1.04	-
Ductility (25 °C, 5 cm/min)	AASHTO T 51 [41]	cm	>100	>100
Flashpoint (Cleveland open cup),	AASHTO T 48 [42]	°C	316	>230

**Table 2:** Performance grading (rheological properties) of asphalt binder (PG 70-16).

Asphalt Binder	Properties	Unit	Test Temperature (°C)	Result	AASHTO M320 [43] Requirements
Original	Flash Point	(°C)	-	316	230 °C, min
	Rotational Viscosity	(mPa.s)	135	745	3000 mPa.s, max
	DSR, G*/sinδ (10 rad/s)	(kPa)	58	7.34	1.00 kPa, min
			64	3.169	
			70	1.453	
			76	0.703	
RTFO- Aged	Mass Loss	(%)	-	0.014	1%, max
	DSR, G*/sinδ (10 rad/s)	(kPa)	64	6.234	2.2 kPa, min
			70	3.138	
			76	1.682	
PAV- Aged	DSR, G*/sinδ (10 rad/s)	(kPa)	25	5145	5000 kPa, max
			28	3386	
	BBR, Creep Stiffness	(MPa)	−16	190	300 MPa, max
	Slope m value	-	-	0.335	0.3, min

**Table 3:** Properties of aggregates.

Material	Designation	Property	Result	Limit
Coarse Aggregates	C 127 [45]	Bulk Specific Gravity	2.580	-
		Apparent Specific Gravity	2.620	-
		Percent Water Absorption	0.42	≤2.0%
	C 131 [46]	Los Angeles Abrasion %	18	≤30%
	D 5821 [47]	Crushed Faces %	96	≥95%
	C 88 [48]	Soundness (Na_2_SO_4_, %)	3.1	≤12%
Fine Aggregates	C 128 [49]	Bulk Specific Gravity	2.60	-
		Apparent Specific Gravity	2.622	-
		Percent Water Absorption	0.6	≤2.0%
	D 2419 [50]	Sand Equivalent %	55	≥50–45%

**Table 4:** Properties of nanomaterials.

Nanomaterial	Chemical Formula	Given Designation	Physical Form	Particle Size (nm)	Density (g/mL)	Specific Surface Area (m^2^/g)	Purity (%)	Unit Price Per 1 Kg (USD)
Nano-Silica	SiO_2_	NS	White Powder	25–35	0.080	190–250	99.8	26.8
Nano-Alumina	Al_2_O_3_	NA		10–20	0.200	120–160	99.9	28.5
Nano-Titanium	TiO_2_	NT		20–30	0.510	120–160	99.9	28.5
Nano-Zinc	ZnO	NZ		15–20	0.331	30–60	99.9	39.4
Carbon Nanotubes	MWCNT	CNT	Black Powder	20 nm dia. 10 μm len.	0.126	100–300	>95	91.5

**Table 5:** Asphalt modification dosages.

Nanomaterial	Reference	Previous Investigated Dosage (%)	Selected Dosages
Nano-Silica	[51]	1, 3, 5	2%, 4%, 6%, 8%, 10%
	[35]	4, 6, 15	
	[27]	2, 4, 6, 8	
Nano-Alumina	[16]	3, 5, 7	
	[26]	3, 6, 9, 12	
	[52]	2, 3, 4, 5	
Nano-Titanium	[53]	1, 2, 5, 10	
	[54]	1, 3, 5, 7	
	[28]	1, 3, 5, 7	
Nano-Zinc	[55]	1, 2, 3, 4, 5	1%, 2%, 3%, 4%, 5%
	[56]	1, 3	
	[57]	1, 3, 5	
Carbon Nanotubes	[58]	1, 2, 3	
	[13]	0.2, 0.4, 0.6, 0.8, 1, 1.5, 3	
	[31]	0.5, 1, 1.5, 2	

**Table 6:** Sulfoxide and carbonyl results.

Material	Dosage %	Sulfoxide Index	Secondary Sulfoxide Index	Carbonyl Index
Binder (NEAT)	0	0.0520	0.0326	0.0135
Nano-Silica (NS)	6	0.0511	0.0292	0.0131
	8	0.0481	0.0240	0.0062
	10	0.0487	0.0225	0.0071
Nano-Alumina (NA)	6	0.0356	0.0319	0.0153
	8	0.0356	0.0312	0.0157
	10	0.0234	0.0262	0.0118
Nano-Titanium (NT)	6	0.0258	0.0288	0.0115
	8	0.0237	0.0265	0.0111
	10	0.0224	0.0253	0.0103
Nano-Zinc (NZ)	3	0.0409	0.0370	0.0129
	4	0.0402	0.0401	0.0133
	5	0.0395	0.0346	0.0119
Carbon Nanotubes (CNTs)	3	0.0429	0.0330	0.0151
	4	0.0345	0.0294	0.0151
	5	0.0403	0.0288	0.0164

**Table 7:** Permanent microstrain at 5000 cycles.

Nanomaterial	Test 1	Test 2	Mean (με)	Std. Dev.	C.O.V. (%)
NS-6	17,500	17,730	17,615	163	0.92
NS-8	4250	4510	4380	184	4.2
NS-10	6780	7028	6904	175	2.54
NA-6	7740	7976	7858	167	2.12
NA-8	15,180	15,430	15,305	177	1.16
NA-10	15,320	15,574	15,447	180	1.16
NT-6	15,220	15,464	15,342	173	1.12
NT-8	14,510	14,754	14,632	173	1.18
NT-10	21,850	22,106	21,978	181	0.82
NZ-3	16,050	16,286	16,168	167	1.03
NZ-4	7220	7464	7342	173	2.35
NZ-5	6130	6382	6256	178	2.85
CNT-3	14,840	15,082	14,961	171	1.14
CNT-4	12,660	12,906	12,783	174	1.36
CNT-5	17,817	24,065	20,941	4418	21.1
NEAT	28,280	28,528	28,404	175	0.62

**Table 8:** Analysis of variance (ANOVA) for permanent strain at 5000 cycles.

Source of Variation	SS	df	MS	F	*p*-Value	F Crit
Between Groups	924,561,785.7	5	184,912,357	5958.44375	5.35616 × 10^−11^	4.387374
Within Groups	186,202	6	31,033.6667			
Total	924,747,987.7	11				

## Data Availability

The original contributions presented in this study are included in this article. Further inquiries can be directed to the corresponding authors.
